# Supplementary material for: The causal relationship between the human gut microbiota and pyogenic arthritis: a Mendelian randomization study
Source: Front Cell Infect Microbiol. 2024 Nov 26;14:1452480. doi: 10.3389/fcimb.2024.1452480 (PMC11629706; doi:10.3389/fcimb.2024.1452480)
Supplement: Supplementary file 1 [file DataSheet1.zip › mendelian test/ebi-a-GCST90027780.csv_forest.pdf]

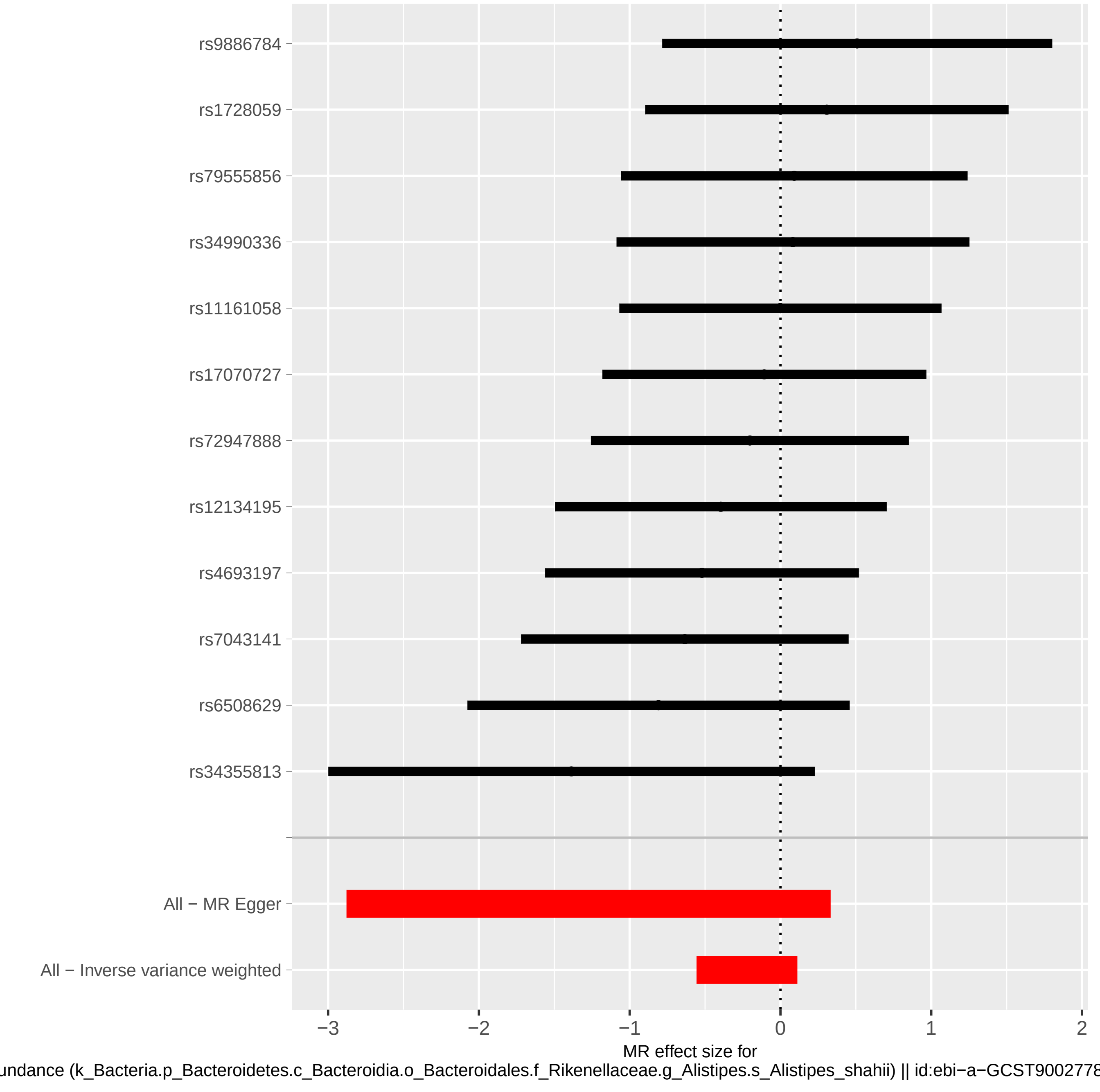
rs9886784
rs1728059
rs79555856
rs34990336
rs11161058
rs17070727
rs72947888
rs12134195
rs4693197
rs7043141
rs6508629
rs34355813
All − MR Egger
All − Inverse variance weighted
−3
−2
−1
0
1
2
MR effect size for
undance (k_Bacteria.p_Bacteroidetes.c_Bacteroidia.o_Bacteroidales.f_Rikenellaceae.g_Alistipes.s_Alistipes_shahii) || id:ebi−a−GCST900277
